# Despotic Leadership and Thriving at Work Among Nurses in a Saudi Tertiary Hospital: A Cross‐Sectional Study

**DOI:** 10.1155/jonm/4697738

**Published:** 2026-10-29

**Authors:** Shaimaa Mohamed Elghareeb Allam, Maryam Murdhi Attalh Alanizi, Amany Ahmed Abdrbo, Rasha Awad Abdelmagied, Sara El Sayed Abd El Atty, Farida Said Abed El Maksoud Ahmed, Somaya Ahmed Bayoumy, Naiema Mohamed Eleseawy Abdullah, Heba Mohammed Mahmoud Elhapashy

**Affiliations:** ^1^ Medical-Surgical Nursing Department Faculty of Nursing Mansoura University Mansoura Egypt mans.edu.eg; ^2^ Department of Nursing College of Pharmacy, Nursing & Medical Sciences Riyadh Elm University Riyadh Saudi Arabia riyadh.edu.sa; ^3^ King Fahad Medical City Riyadh Saudi Arabia kfmc.med.sa; ^4^ Nursing Management and Informatics Department of Nursing College of Pharmacy, Nursing & Medical Sciences Riyadh Elm University Riyadh Saudi Arabia riyadh.edu.sa; ^5^ Adult Health Care Nursing Department Faculty of Nursing Capital & Innovation University Helwan Egypt; ^6^ Nursing Administration Faculty of Nursing Cairo University Cairo Egypt cu.edu.eg; ^7^ Nursing Administration Faculty of Nursing Badr University Cairo Egypt; ^8^ Community Health Nursing Faculty of Nursing Capital University Helwan Egypt capital.edu

**Keywords:** despotic leadership, leadership, occupational well-being, Registered nurses, Saudi Arabia, tertiary healthcare hospital, thriving at work

## Abstract

**Background:**

Despotic leadership is characterized by controlling, self‐serving, and unethical leadership behaviors, whereas thriving at work reflects positive work‐related well‐being and professional functioning. However, empirical evidence on the relationship between perceived despotic leadership and nurses’ thriving at work in Saudi tertiary healthcare settings remains limited.

**Aim:**

To examine the association between perceived despotic leadership and thriving at work among registered nurses in a Saudi tertiary hospital.

**Methods:**

We conducted a quantitative, cross‐sectional correlational study at King Fahd Medical City in Riyadh, Saudi Arabia, using a convenience sample of 181 registered nurses with at least 12 months of experience in the organization. Data were collected using a demographic and professional information form, the 6‐item Despotic Leadership Scale, and the 30‐item long form of the Thriving from Work Questionnaire. We used descriptive statistics, independent‐samples *t* tests, one‐way analysis of variance, Pearson correlation, item‐level correlations, and multivariable linear regression for analysis.

**Results:**

Nurses reported moderate levels of perceived despotic leadership (mean = 3.19 ± 1.21) and thriving at work (mean = 3.29 ± 1.05). Perceived despotic leadership was significantly and negatively associated with thriving at work (*r* = −0.323, 95% CI [−0.448, −0.186], *p* < 0.001). In the adjusted analysis, perceived despotic leadership remained significantly associated with lower thriving at work (*B* = −0.321, SE = 0.061, *β* = −0.371, 95% CI [−0.441, −0.201], *p* < 0.001).

**Conclusion:**

Higher perceived despotic leadership was associated with lower thriving at work among registered nurses. Because the study was cross‐sectional, causal inferences cannot be made. The findings support organizational efforts to strengthen ethical, supportive, and participatory leadership practices and warrant longitudinal research to clarify the direction of this relationship.

## 1. Introduction

Increasingly, healthcare organizations are acknowledging the importance of leadership practices as core drivers of the psychological well‐being of nurses, their professional performance and, importantly, their retention in the workforce, as well as the quality of patient care. With the ongoing nursing shortages, the increased acuity of their patients, and the growing demands for healthcare services, identifying the types of leadership behaviors that support or impede nurses’ ability to thrive is becoming an essential part of healthcare organizations’ strategic focus. In this context, examining the effects of destructive leadership styles, particularly despotic leadership, is becoming increasingly important in healthcare organizations [1].

Despotic leadership in health care is characterized by a leadership style that leaves staff with no opportunity to influence decisions; they can be controlled through methods such as threats or intimidation [2].

It is common knowledge today that dictators require and impose strict compliance from their followers. Despotic leaders inhibit new and different ways of working (such as teamwork and creative thinking) and create an atmosphere of fear, low morale, and little or no communication [3]. Despotic leaders contribute to the deterioration of the work environment and ultimately affect the productivity of staff and the quality of the care received by patients [4].

In contrast, thriving at work means being in a positive mental state, which embodies both feeling vital and learning. Successful nurses are energized, engaged, and growing, which enables resilience, adaptability, and having an impact on patients [1]. Thriving promotes job satisfaction, dedication, collaboration, and innovation, as well as protects nurses against burnout and disengagement. When well‐being thrives, organizations enjoy the success of higher‐quality care, patient safety, performance, and reduced turnover. As such, despotic control stifles nurse potential, whereas thriving facilitates development, cooperation, and overall enhancement of healthcare services [5].

In healthcare organizations, leadership behaviors are especially influential because nurses work in complex environments that require continuous communication, rapid decision‐making, emotional resilience, and professional collaboration. When nurse managers adopt despotic or highly controlling behaviors, nurses may experience reduced autonomy, limited participation in clinical decision‐making, emotional exhaustion, and organizational silence, all of which may negatively affect their professional functioning and psychological well‐being [3]. Such conditions can weaken nurses’ sense of psychological safety and may prevent them from developing the vitality and learning that are central to thriving at work [1].

Therefore, examining the relationship between despotic leadership and thriving at work among nurses matters because it clarifies how negative leadership practices may affect not only nurses’ well‐being but also the broader quality, safety, and sustainability of healthcare services [6]. Previous studies have explored the impacts of destructive leadership, but little has examined how despotic leadership affects registered nurses in Saudi Arabia in their ability to thrive in their workplace. Therefore, this paper will investigate this relationship in light of the above findings.

### 1.1. Problem Statement

Nurses have a major responsibility for maintaining patient safety and providing quality care; however, they may work in environments characterized by negative leadership practices. Despotic leaders exhibit controlling, self‐serving, and authoritarian behaviors that may be associated with lower autonomy, emotional exhaustion, and organizational silence. Although previous studies have linked despotic leadership with adverse nurse and workplace outcomes, evidence specifically examining its relationship with nurses’ ability to thrive at work remains limited, particularly in Saudi Arabia [7, 8].

Thriving at work captures positive work‐related functioning and well‐being. Despite evidence linking destructive leadership with adverse employee outcomes, relatively little research has examined the empirical relationship between perceived despotic leadership and nurses’ thriving at work in Saudi healthcare settings [1, 5, 9]. Therefore, this study aimed to examine the association between perceived despotic leadership and thriving at work among registered nurses at King Fahd Medical City (KFMC) in Riyadh, Saudi Arabia.

### 1.2. Theoretical Framework

The current research is based on two mutually supportive theories, which are as follows: Conservation of resources (COR) theory and self‐determination theory (SDT). Specifically, COR theory states that undesirable work conditions may put either psychological or energy resources at risk. On the other hand, SDT focuses on how work conditions may either help satisfy or impede the basic psychological needs.

In COR theory, people are always trying to obtain, protect, and preserve important resources, and the presence of stress occurs whenever the resources are in danger, lost, or not restored enough [10, 11]. In nursing work, controlling, self‐serving, and punishing leadership behaviors are perceived as a threat to resources since they limit access to support, acknowledgment, predictability, voice, and emotional energy. Nurses may therefore spend a lot of resources to deal with stress from work and have fewer resources left to perform in a positive way at work and to develop professionally [12, 13].

SDT enhances the resource‐based rationale by emphasizing the type of motivation and psychological needs satisfied. According to SDT, autonomy, competence, and relatedness are the basic requirements needed for optimal motivation and well‐being [14]. Tyrannical leadership can inhibit autonomy by restricting employees’ participation in decisions; competence by destroying confidence or providing negative feedback; and relatedness by disrupting positive and supportive relationships in the workplace [15, 16].

Taken together, COR theory and SDT suggest complementary mechanisms rather than competing explanations. COR theory emphasizes the potential loss or depletion of valued work resources, while SDT emphasizes the frustration of basic psychological needs. Both processes are theoretically inconsistent with a work environment that supports nurses’ positive functioning and development. Accordingly, the present cross‐sectional study tested the expectation that higher perceived despotic leadership would be associated with lower thriving at work; it did not test causal pathways or mediation mechanisms.

Conceptually, perceived despotic leadership was treated as the independent study variable and thriving at work as the outcome variable. Consistent with the cross‐sectional design, these labels describe the analytic model and do not imply temporal precedence or causation.

### 1.3. Significance of the Study

This study intends to close a significant void in the body of research on the effects of autocratic leadership on nurses’ psychological growth and career success, to contribute to the stability of nursing as a profession [7]. Additionally, it will provide nurse managers, organizations, and public policymakers with empirical evidence that can be utilized when developing effective leadership methods for creating an environment that will support nurses’ well‐being, increase employee engagement, and provide avenues for continuing education or development [6]. Therefore, the conclusion draws the connection that positive leadership styles create positive outcomes such as improved retention rates for nurses, improved patient safety, and improved quality of care delivered through the health care system.

### 1.4. Aim of the Study

To examine the association between perceived despotic leadership and thriving at work among registered nurses.

Objectives of the study:

1. To assess the level of perceived despotic leadership behaviors among nurses
2. To assess the level of thriving at work among nurses
3. To examine the relationship between despotic leadership and thriving at work among nurses.

Research Questions:

1. To what extent do registered nurses perceive their immediate nurse managers as exhibiting despotic leadership behaviors?
2. To what extent do registered nurses experience thriving at work?
3. What is the relationship between perceived despotic leadership and thriving at work among registered nurses?

## 2. Methods

### 2.1. Research Design

Data collection for this study was conducted over a period of 4 weeks (April 15–May 15, 2026) using the same self‐administered survey to collect the quantitative data from all participants. A quantitative cross‐sectional correlational design was used to assess the relationship between perceived despotic leadership and their ability to thrive at work. As a result, the study design allowed the assessment of relationships between multiple variables at one point in time in the study, rather than by manipulating the environment within the context of the study.

### 2.2. Setting

#### 2.2.1. Setting

The study was conducted at the Main Hospital of KFMC, a tertiary healthcare complex located in Riyadh, Saudi Arabia. According to the official KFMC at a Glance publication, the Main Hospital had a reported capacity of 235 beds, while the entire medical city had a total capacity of approximately 1200 beds [17]. The Main Hospital provides medical, surgical, critical care, and dental services. The present study was conducted exclusively at the Main Hospital. KFMC provides a wide range of medical services such as acute care, urgent care, short‐stay surgical services, maternal services, and numerous highly specialized outpatient services. Such diversity among medical environments makes KFMC an excellent case study for examining leadership across a variety of clinical settings. KFMC provides a wide range of medical services such as acute care, urgent care, short‐stay surgical services, maternal services, and numerous highly specialized outpatient services. Such diversity among medical environments makes KFMC an excellent case study for examining leadership across a variety of clinical settings.

#### 2.2.2. Study Population

The study population included all the registered nurses working directly with patients at KFMC in Riyadh. Registered nurses were eligible if they provided direct care within an inpatient medical or surgical unit, critical care area, inpatient or outpatient emergency department, operating room, women’s and children’s services, or any other area of specialization. All eligible subjects had been employed by the hospital for a minimum of 1 year. According to statistical records provided by the human resources (HR) department, approximately 340 registered nurses were working at the Main Hospital of KFMC during the study period. This figure represents the nursing workforce of the selected hospital rather than the entire medical city. The accessible study population was drawn from these nurses based on the study’s eligibility criteria.

### 2.3. Sampling Technique

The research for this study used a convenience sampling approach to engage participants. Eligible clinical nurses who satisfied the inclusion requirements and were accessible at the time of data gathering were recruited to participate. The researcher made a concerted effort to obtain the participation of a wide variety of nurses from numerous clinical departments in order to enhance the diversity and representativeness of the participant sample.

#### 2.3.1. Eligibility Criteria

Inclusion and exclusion criteria were developed to have a well‐qualified participant sample with positive exposure to nurse manager leadership practices and the clinical work environment they manage.

#### 2.3.2. Inclusion Criteria

• Registered nurses who are employed at KFMC, Riyadh, Saudi Arabia
• Registered nurses who are actively engaged in direct patient care
• Registered nurses who have at least 1 year of clinical experience with the organization
• Registered nurses who are working in either an inpatient or outpatient clinical department during the data collection period
• Registered nurses who voluntarily agree to participate in this research study
• Nurses of nationalities, levels of education, and titles who are not involved in any other clinical nursing practice are eligible for inclusion.

#### 2.3.3. Exclusion Criteria

• Nursing administrators, interns, and students in clinical training
• Nurses on annual/maternity/sick/long‐term leave during data collection, and those who declined to participate or submitted incomplete questionnaires

### 2.4. Sample Size Determination

The Krejcie and Morgan [18] sample size determination method for finite populations provides a sample size based on an accessible population of 340 registered nurses using a method that allows for a 95% confidence level, 5% margin of error, and 50% response distribution, with a minimum sample size of 181 participants. Based on this calculation, the minimum required sample size was set at 181 registered nurses. The final sample included 181 eligible nurses, meeting the calculated sample size requirement.

The minimum required sample size was estimated at 181 nurses using the Krejcie and Morgan [18] method. Of the 217 nurses who received the survey invitation, 19 participated in the pilot study and 17 were excluded due to incomplete responses, ineligibility, or declining participation. The remaining 181 eligible nurses completed the questionnaire and were included in the final analysis. Thus, the final sample coincided with the calculated minimum sample size. The participant selection process is illustrated in Figure 1.

**FIGURE 1:**
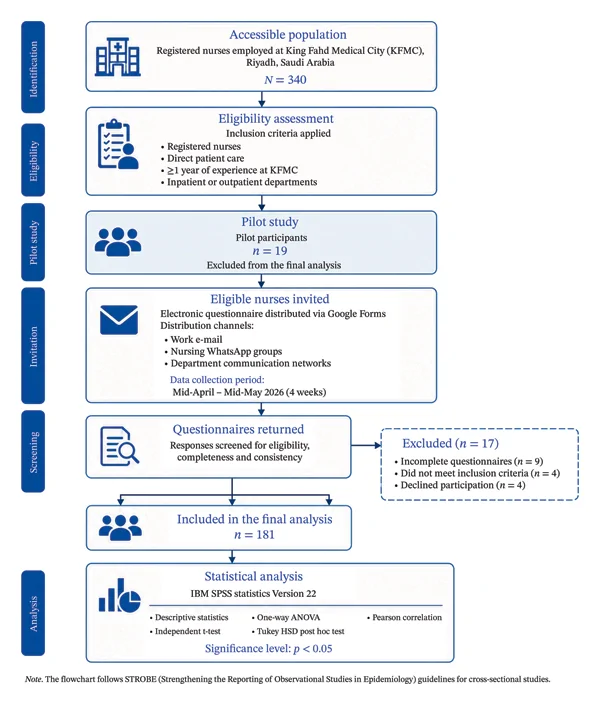
Flow diagram of participant recruitment and selection process.

### 2.5. Data Collection Instruments

Data were collected using a structured questionnaire consisting of the following three tools:

#### 2.5.1. Tool I: Demographic and Professional Information Form

Participants completed a demographic and professional information form developed by a researcher to gather information about their personal and work characteristics. This form included questions intended to assess participant age, gender, marital status, educational level, nursing experience, department or clinical unit they work in, average weekly working hours, employment status, work schedule pattern, current job title, and average number of patients seen during one shift. These variables were assessed in order to better describe the study sample as well as to explore possible differences in perceptions of tyrannical leadership or thriving in the workplace based on different demographic and professional characteristics.

#### 2.5.2. Tool II: Despotic Leadership Scale

The Despotic Leadership Scale, developed by De Hoogh and Den Hartog [8], was used to assess nurses’ perceptions of despotic, self‐serving, and unethical behaviors exhibited by their immediate nurse managers. The instrument consists of six items rated on a five‐point Likert scale ranging from 1 (strongly disagree) to 5 (strongly agree).

All six items are scored in the same direction, with higher item scores reflecting greater perceived despotic leadership; therefore, no reverse‐coded items are required. The total score is calculated by summing the responses to all six items, yielding a possible range of 6–30. The overall mean score is obtained by dividing the total score by six, producing a mean ranging from 1 to 5. Higher total and mean scores indicate greater perceived despotic leadership, whereas lower scores indicate less perceived despotic leadership.

Based on the percentage mean scores, perceived despotic leadership was classified as low (less than 60%), moderate (60% to less than 75%), or high (75% and above). For this classification, the percentage mean score is calculated by dividing the overall mean score by the maximum possible item score (5) and multiplying by 100. These categories were used for descriptive interpretation rather than as established diagnostic cutoffs.

The Despotic Leadership Scale [8] has been used in previous studies and shown to have good internal consistency (Cronbach’s alpha ranging from 0.82 to 0.88). It has been extensively used in organizational studies to measure the despotic and self‐serving aspects of leadership behavior, with good evidence of its reliability and construct validity in various cultural and occupational contexts.

#### 2.5.3. Tool III: Thriving at Work Scale

Nurses’ work‐related well‐being was assessed using the 30‐item long form of the Thriving at Work Questionnaire, developed by the Harvard T.H. Chan School of Public Health, Center for Work, Health, and Well‐being [19]. The questionnaire was developed through a multiphase process involving expert consultation, cognitive interviewing, reliability testing, and psychometric validation. The instrument assesses six domains of thriving from work: psychological and emotional well‐being, social well‐being from work, work–life integration, job design and experience of work, basic needs for thriving, and physical and mental well‐being.

The 30‐item long form was selected to provide a comprehensive assessment of work‐related thriving across these domains. In the present study, items were administered using an adapted five‐point Likert‐type scale ranging from 1 (strongly disagree) to 5 (strongly agree). According to the scoring criteria of the initial tool, a total of two negatively worded statements must be reversed: (1) item 28—“The amount of stress from my work is too high,” and item 29—“I am concerned about being in danger at my workplace.” With a five‐point scale employed in this study, the reverse coding component has been defined using the following formula: (6—initial item score). For the means of the full questionnaire, the average thriving status has been measured by adding the scores of 28 directly scored items and two reverse‐coded statements and dividing this sum by 30. The obtained result is in the range of 1–5, where a higher score means a bigger thriving rate. Overall percentages of mean scores have been classified as follows: less than 60%—low scores, 60% to less than 75%—moderate scores, more than or equal to 75%—high scores.

In the past, psychometric assessment revealed good construct validity and internal reliability of the original questionnaire [20]. Nevertheless, the original questionnaire prescribes a six‐category frequency response format and scoring by means of summation. That is why, the agreement scale used in this paper and computed average scores can be considered adaptations of the original questionnaire that need to be taken into account when interpreting the results and comparing them with the findings obtained in other studies.

### 2.6. Instrument Validation and Reliability

#### 2.6.1. Face Validity

The English versions of the questionnaires were translated into Arabic using the forward–backward method (English–Arabic–English), in order to verify linguistic accuracy and conceptual equivalence. The Arabic questionnaires were then reviewed by 11 experts in nursing administration, nursing research, and clinical practice to evaluate the questionnaires with regard to clarity, understanding, and cultural relevance. The changes suggested by the experts were implemented in the Arabic questionnaires.

According to experts’ suggestions, slight changes in wording and layout were applied to ensure better understanding by the target audience. The demographic and occupational information form was updated so that it includes work status, work schedule type, occupation title, mean working hours per week, and average number of patients treated per shift. These changes were added to the final version of the questionnaire before data collection.

#### 2.6.2. Content Validity

A panel of expert evaluators evaluated each item based on a variety of factors, including content validity. Content validity was established through panel members’ assessment of the item’s information as it related to the study purpose and design. Based on their assessment, the total number of items assessed for content validity resulted in an overall scale content validity index (S‐CVI) of 0.96, indicating excellent content validity and demonstrating that the instruments were highly appropriate for assessing perceived despotic leadership and thriving at work among nurses. After incorporating the experts’ feedback, the final version of the questionnaire was approved for use in the study.

#### 2.6.3. Reliability

In the present study, Cronbach’s alpha (*α*) provided an assessment of the internal consistency reliability of those study measures. Acceptable, reliable measures had a Cronbach’s alpha of ≥ 0.70. Both Tool II (Despotic Leadership Scale) and Tool III (Thriving at Work Scale) exhibited strong (*α* = 0.82) and excellent (*α* = 0.89) internal consistency reliability, respectively, indicating both tools provided reliable measurements of study variables in nurses who participated in this research.

#### 2.6.4. Pilot Study

The purpose of conducting a pilot study is to test whether a specific research instrument performs adequately for the research goal. This can be done effectively with a small number of people (10%), 19 registered nurses who met the criteria to participate in the larger study. The pilot study was designed to evaluate the clarity, usability, feasibility, and understanding of the research instruments and to estimate the time needed to complete the online portion of the survey. Data collected from the pilot study were excluded from the final analysis.

#### 2.6.5. Procedures of the Study

Before conducting this study, we reviewed current literature on destructive leadership, the negative effects of destructive leadership on Nursing environments and nurse mental health, to gather existing knowledge and create a foundation for our own research [21]. After obtaining all necessary Institutional Review Board (IRB) approvals, administrative approvals within the Riyadh Second Health Cluster, Executive Administration for Nursing Affairs, Nursing Research Committee, our study was conducted over 4 weeks (mid‐April to mid‐May, 2026) through registered nurses who met predetermined criteria based on their eligibility according to the inclusion and exclusion requirements established by the nursing administrators and unit managers of the clinical departments participating in the study.

The investigation had several components that included gathering demographic and professional data from the nurses, using the Despotic Leadership Scale and Thriving at Work Scale. The data were collected electronically, using a Google Form that was made available in Arabic and English to participants through official communication channels. The survey link was sent out via work email, departmental communication systems, and WhatsApp Groups for nurses. Before accessing the survey, participants received information on the purpose of the study, research procedures, that all participation was voluntary, how information would be protected, and how they could withdraw from the study without any consequences. Informed consent from participants was obtained electronically and was a necessary step in Google Forms. Before they received the survey link, subjects had to agree to participate. A total of 181 nurses took part in the study and completed the questionnaire, and their results were used for statistical analysis. However, it was impossible to establish how many respondents had opened the given link. To avoid duplicate responses, the online questionnaire was set up in Google Forms in a way that allowed only one answer per participant via logging in with Google account details.

The questionnaire required roughly 10–15 min to fill out. A total of 217 nurses were sent the invitation for the electronic survey via their work emails, WhatsApp groups of nurses, and departmental channels. From this group, 19 nurses took part in the pilot study and were removed from the final analysis. Furthermore, 17 individuals were excluded: 9 because the questionnaire was not filled out completely, 4 who did not meet the requirements, and 4 who refused to participate. Thus, the final analysis incorporated 181 eligible nurses, as shown in Figure 1.

In order to boost participation rate in the process of data collection, reminder messages were sent via the same communication medium during the process. Once the data collection finished, the online survey tool was shut down, and all responses were analyzed for completeness and eligibility. During the data analysis, if any response was either incomplete or illegible, it was removed from the process. The data were coded, thereby ensuring data accuracy.

### 2.7. Statistical Analysis Plan

The collected data were coded, categorized, scored, tabulated, and analyzed using SPSS version 22.0 [22]. The Despotic Leadership Scale consists of six items that were all scored in a constant direction, and none of the items were reverse‐coded. The final mean score was achieved by calculating the sum of the six original scores and dividing it by six. In relation to the Thriving from Work Questionnaire consisting of 30 items, two negatively worded items, which are Items 28 and 29, had to be reverse‐coded using the following formula (6 − original scoring). Finally, the mean score was achieved by adding the 30 items scored appropriately and dividing by 30.

Data were evaluated to check completeness, identify disregarded points, and ensure approximate normality by looking at the skewness and kurtosis statistics. Summary of participant characteristics and study measures was done through frequencies, percentages, means, and standard deviations. The independent‐samples *t* test was employed for the two‐sample comparison, while the one‐way analysis of variance (ANOVA) was done to compare the mean score between variables with regard to the demographic and occupational factors. However, the reliability of the comparison was limited due to the small number of respondents referred to in some categories.

Therefore, the results concerning small subgroups were interpreted with caution, and their statistical significance should be regarded as provisional until the pertinent assumptions have been verified. Pearson’s correlation coefficient (r) was calculated to investigate the connection between perceptions of despotic leadership in general and the concept of thriving at work, and a 95% confidence interval was determined for the correlation using Fisher’s r‐to‐z transformation. Subsequently, more Pearson correlations were performed for each despotic leadership statement in relation to the general thriving at work score. Then, multiple linear regression was utilized to examine the relationship between perceptions of despotic leadership and thriving at work while controlling for gender, years of experience in nursing, department/unit, average working hours per week, and work pattern. Categorical covariates were introduced as appropriate. Two‐tailed tests were applied, and the significance level was 0.05. As it is a cross‐sectional study, the results are interpreted in terms of association only.

## 3. Results

Table 1 reveals personal characteristics among participating nurses. Regarding age, the mean age was (34.45 ± 8.22), indicating that the sample mainly represented adult nurses in the early to middle stages of their professional careers. Regarding gender, females constituted the majority of the sample (63.0%). Concerning marital status, the highest proportion of nurses were married (60.8%). In terms of educational level, the majority of participants held a bachelor’s degree in nursing (87.8%). Regarding years of nursing experience, the highest percentage of nurses had more than 10 years of experience (32.6%). As for the department/unit of work, the outpatient department showed the highest representation (26.5%). Concerning average weekly working hours, most nurses worked 40–49 h per week (82.3%). Regarding employment status, the majority of participants were full‐time employees (96.7%). In terms of work schedule patterns, rotating shifts were the most common (54.1%). Finally, the number of working hours per week on average was (44.04 ± 9.30). In addition, the mean number of patients assigned per shift was (25.04 ± 8.30).

**TABLE 1:** Distribution of the study participants according to their socio‐demographic and professional characteristics (*n* = 181).

Personal characteristics	*n*	%
Age (years) mean ± SD	34.45 ± 8.22	
Gender		
Male	67	37
Female	114	63
Marital status		
Married	110	60.8
Divorced	7	3.8
Single	63	34.8
Widowed	1	0.6
Level of education		
Diploma in Nursing	10	5.5
Bachelor’s Degree in Nursing	159	87.8
Master’s & Doctorate Degree (PhD/DNP)	12	6.6
Work experience as a nurse		
1–2 years	22	12.2
3–4 years	36	19.8
5–7 years	24	13.3
8–10 years	40	22.1
More than 10 years	59	32.6
Department/unit		
Medical ward	33	18.2
Surgical ward	13	7.3
Intensive care unit (ICU)	14	7.7
Emergency department	12	6.6
Maternity/obstetrics	7	3.9
Pediatric unit	4	2.2
Operating room	7	3.9
Outpatient	48	26.5
Department dialysis unit	6	3.3
Other	37	20.4
Average weekly working hours		
Less than 20 h	21	11.6
20–29 h	0	0
30–39 h	1	0.6
40–49 h	149	82.3
50 h or more	10	5.5
Employment status		
Full‐time	175	96.7
Part‐time	4	2.2
Other	2	1.1
Work schedule pattern:		
Fixed day shift	76	42.0
Rotating shift	98	54.1
Fixed night shift	6	3.3
On‐call system	1	0.6

	**Mean ± SD**	

How many hours do you work per week on average	44.04 ± 9.30	
On average, how many patients are assigned per shift	25.04 ± 8.30	

Table 2 indicates that the overall mean score of nurses’ perceptions of despotic leadership was 3.19 ± 1.21, indicating a moderate level of perceived despotic leadership. The highest mean score was reported for “My leader takes full credit for successes and shifts blame for failures onto others” (3.31 ± 1.31), followed by “My leader uses charm or charisma primarily for self‐benefit” (3.28 ± 1.33). The lowest mean score was observed for “My leader behaves in an authoritarian and dictatorial manner” (3.02 ± 1.35), followed by “My leader expects complete and unquestioning obedience from subordinates” (3.04 ± 1.32).

**TABLE 2:** Nurses’ perception of despotic leadership (*n* = 181).

Despotic leadership items	Mean ± SD
My leader behaves in an authoritarian and dictatorial manner	3.02 ± 1.35
My leader expects complete and unquestioning obedience from subordinates.	3.04 ± 1.32
My leader treats employees harshly and shows little compassion.	3.25 ± 1.33
My leader manipulates others to serve personal interests.	3.25 ± 1.38
My leader uses charm or charisma primarily for self‐benefit.	3.28 ± 1.33
My leader takes full credit for successes and shifts blame for failures onto others.	3.31 ± 1.31

Overall mean score	3.19 ± 1.21

*Note:* Responses were rated on a 5‐point Likert scale ranging from 1 (strongly disagree) to 5 (strongly agree).

Abbreviation: SD = standard deviation.

Table 3 shows that the overall mean score of thriving at work among participating nurses was (3.29 ± 1.05), indicating a moderate level of thriving. The highest mean score was reported for two items: “My work adds meaning to my life” (3.51 ± 1.28) and “I am treated with respect at work” (3.48 ± 1.23), followed by “I feel safe getting to and from work” (3.45 ± 1.25) and “I can achieve a healthy balance between my work and my life outside of work” (3.45 ± 1.25). On the other hand, “After I leave work, I have enough energy to do the things I want or need to do” was reported as the lowest mean score (2.96 ± 1.29), followed by “I worry that I will get hurt at work” (3.12 ± 1.27) and “I am satisfied with the amount of paid leave I can take to care for myself or family members” (3.14 ± 1.26).

**TABLE 3:** Nurses’ thriving at work (*n* = 181).

Thriving at work items	Mean ± SD
My work adds meaning to my life (meaning)	3.51 ± 1.28
My job allows me to achieve my full potential (potential).	3.36 ± 1.18
The kind of work I do makes me happy (happiness)	3.34 ± 1.19
I love my job (enthusiasm)	3.42 ± 1.23
I am satisfied with my job (satisfaction)	3.41 ± 1.2
My work adds to my overall life satisfaction (life satisfaction).	3.43 ± 1.25
I feel supported by the people I work with (support)	3.33 ± 1.2
I feel valued by the people I work with (valued).	3.35 ± 1.2
I am treated fairly at work (fairness).	3.35 ± 1.24
I am treated with respect at work (respect).	3.48 ± 1.23
At work, I feel like I belong (belonging)	3.36 ± 1.2
I receive recognition at work for my accomplishments (recognition)	3.33 ± 1.18
I can voice concerns or make suggestions at work without getting into trouble (voice).	3.34 ± 1.23
I can easily manage my job as well as attend to the needs of my family (work–family)	3.28 ± 1.24
I feel safe getting to and from work (commute)	3.45 ± 1.25
I can achieve a healthy balance between my work and my life outside of work (work–life)	3.45 ± 1.25
I am paid fairly for the job I do (pay fairness).	3.24 ± 1.22
I am satisfied with the amount of paid leave I can take to care for myself or family members (benefits)	3.14 ± 1.26
I feel my job is secure (job security)	3.22 ± 1.19
I have good promotion opportunities (progression)	3.30 ± 1.24
I am happy with how much input I have in decisions that affect my work (autonomy)	3.20 ± 1.20
I have adequate control over the pace of my work (work intensity)	3.24 ± 1.19
I am happy with how much control I have over my work schedule (schedule)	3.26 ± 1.23
I can easily manage the demands of my job (demands)	3.24 ± 1.22
I have access to the resources I need to do my job well (resources)	3.31 ± 1.16
I feel physically safe at work (physical safety)	3.36 ± 1.28
I feel psychologically safe at work (psychological safety)	3.34 ± 1.25
I feel excessive levels of stress from my work (stress)	3.24 ± 1.24
I worry that I will get hurt at work (injury)	3.12 ± 1.27
After I leave work, I have enough energy to do the things I want or need to do (energy)	2.96 ± 1.29

Overall mean score	**3.29 ± 1.05**

*Note:* Responses were rated on a five‐point Likert scale ranging from 1 (strongly disagree) to 5 (strongly agree). The bold values represent the overall mean score and standard deviation of nurses’ thriving at work.

Abbreviation: SD = standard deviation.

Table 4 clarifies the differences in nurses’ perceptions of despotic leadership according to their personal characteristics. No statistically significant differences were found between nurses’ personal characteristics and despotic leadership scores (all *p* > 0.05).

**TABLE 4:** Differences in nurses’ perceptions of despotic leadership according to their participants’ personal and professional characteristics (*n* = 181).

Participants’ personal and professional characteristics		Nurses’ perceptions of despotic leadership		*p*
		Mean ± SD	Test statistic	
Age		34.45 ± 8.22	*r* = −0.084	0.261

Gender	Male	3.09 ± 1.26	*t* = −0.786	0.433
	Female	3.24 ± 1.18		

Marital status	Married	3.15 ± 1.15	*F* = 0.521	0.668
	Divorced	2.78 ± 1.32		
	Single	3.28 ± 1.30		
	Widowed	3.83; SD = NA		

Level of education	Diploma in Nursing	3.48 ± 0.97	*F* = 0.712	0.546
	Bachelor’s Degree	3.15 ± 1.24		
	Master’s & Doctorate	2.83 ± 0.45		

Work experience as a nurse	1–2 years	3.07 ± 1.49	*F* = 0.561	0.691
	3–4 years	3.04 ± 1.36		
	5–7 years	3.04 ± 1.10		
	8–10 years	3.2 ± 1.06		
	More than 10 years	3.3 ± 1.15		

Department/unit	Medical ward	3.15 ± 1.32	*F* = 1.880	0.058
	Surgical ward	2.80 ± 1.08		
	Intensive care unit	2.3 ± 1.05		
	Emergency department	3.4 ± 1.49		
	Maternity/obstetrics	2.7 ± 1.12		
	Pediatric unit	3.12 ± 1.57		
	Operating room	3.4 ±0 .74		
	Outpatient department	3.2 ± 1.18		
	Dialysis unit	2.7 ±0 .73		
	Other	3.6 ± 1.07		

Average weekly working hours	Less than 20 h	2.92 ± 1.18	*F* = 0.749	0.524
	30–39 h	2.0; SD = NA		
	40–49 h	3.23 ± 1.20		
	50 h or more	3.28 ± 1.34		

Employment status	Full time	3.17 ± 1.21	*F* = 0.528	0.591
	Part‐time	3.4 ±0 .63		
	Other	4.0 ± 1.41		

Work schedule pattern	Fixed day shift	3.11 ± 1.22	*F* = 0.461	0.710
	Rotating shift	3.2 ± 1.21		
	Fixed night shift	3.3 ± 0.83		
	on‐call system	2.16; SD = NA		

*Note:* Some subgroups had very small sample sizes, including widowed nurses (*n* = 1), nurses working 30–39 h per week (*n* = 1), and nurses on an on‐call schedule (*n* = 1). These subgroup comparisons should be interpreted with caution. SD was not calculated for groups containing only one participant.

Table 5 presents the differences in nurses’ thriving at work according to their personal characteristics. There were highly statistically significant differences regarding department/unit (*F* = 2.859, *p* = 0.004). No statistically significant differences were found according to age, gender, residence, marital status, educational level, years of nursing experience, average weekly working hours, employment status, or work schedule pattern (all *p* > 0.05).

**TABLE 5:** Differences in nurses’ thriving at work according to participants’ personal and professional characteristics (*n* = 181).

Participants’ personal and professional characteristics		Nurses’ thriving at work		*p*
		Mean ± SD	Test statistic	
Age		34.45 ± 8.22	*r* = −0.033	0.662

Gender	Male	3.10 ± 1.09	*t* = −1.884	0.061
	Female	3.4 ± 1.01		

Marital status	Married	3.27 ± 0.99	*F* = 1.036	0.378
	Divorced	2.70 ± 1.46		
	Single	3.39 ± 1.08		
	Widowed	3.83; SD = NA		

Level of education	Diploma in Nursing	3.16 ± 0.91	*F* = 0 0.712	0.546
	Bachelor’s Degree	3.32 ± 1.06		
	Master’s & Doctorate	3.30 ± 0.50		

Work experience as a nurse	1–2 years	3.57 ± 1.13	*F* = 1.797	0.131
	3–4 years	3.42 ± 1.02		
	5–7 years	3.26 ± 1.17		
	8–10 years	2.94 ± 1.06		
	More than 10 years	3.37 ± 0.94		

Department/unit	Medical ward	3.17 ± 1.14	*F* = 2.859	^∗∗^0.004
	Surgical ward	2.89 ± 0.95		
	Intensive care unit	3.66 ± 1.01		
	Emergency department	3.02 ± 1.26		
	Maternity/obstetrics	2.37 ± 1.21		
	Pediatric unit	4.02 ± 0.82		
	Operating room	2.36 ± 0.79		
	Outpatient department	3.62 ± 0.96		
	Dialysis unit	2.98 ± 1.09		
	Other	3.39 ± 0.813		

Average weekly working hours	Less than 20 h	3.46 ± 1.01	*F* = 0.652	0.583
	30–39 h	2.36; SD = NA		
	40–49 h	3.26 ± 1.05		
	50 h or more	3.53 ± 0.98		

Employment status	Full time	3.30 ± 1.05	*F* = 0.955	0.387
	Part‐time	2.74 ± 0.96		
	Other	3.95 ± 0.071		

Work schedule pattern: Rotating shifts	Fixed day shift	3.46 ± 1.08	*F* = 1.192	0.314
	Rotating shift	3.18 ± 1.01		
	Fixed night shift	2.99 ± 1.09		
	On‐call system	3.63; SD = NA		

*Note: r* = Pearson’s correlation coefficient; *t* = independent‐samples *t* test; *F* = one‐way analysis of variance (ANOVA). Some subgroups had very few participants, including widowed nurses (*n* = 1), nurses working 30–39 h per week (*n* = 1), and those on an on‐call schedule (*n* = 1). Therefore, these findings should be interpreted with caution. SD is not applicable for groups with only one participant.

Abbreviation: SD = standard deviation.

^∗∗^ *p* < 0.01.

Table 6 shows the association between perceived despotic leadership and thriving at work among participating nurses. A statistically significant negative correlation was observed (*r* = −0.323, 95% CI [−0.448, −0.186], *p* < 0.001), indicating that higher perceived despotic leadership was associated with lower thriving at work.

**TABLE 6:** Pearson’s correlation between despotic leadership and thriving at work among participating nurses (*n* = 181).

Independent variable	Dependent variable	Pearson’s *r*	*p*‐value	95% CI for *r*
Perceived despotic leadership	Thriving at work	−0.323	< 0.001	−0.448, −0.186

*Note:* Pearson’s correlation coefficients (*r*).

^∗∗^ *p* < 0.001.

Table 7 presents the item‐level correlations between despotic leadership behaviors and thriving at work. All six behaviors were significantly negatively associated with thriving at work. The strongest negative correlation was observed for expecting complete and unquestioning obedience from subordinates (*r* = −0.359, *p* < 0.001), followed by authoritarian and dictatorial behavior (*r* = −0.347, *p* < 0.001). The remaining item‐level correlations ranged from *r* = −0.250 to −0.274 and were also statistically significant.

**TABLE 7:** Correlation of individual despotic‐leadership behaviors with thriving at work.

Despotic leadership behavior	*r* with thriving	*p*‐value
Behaves in an authoritarian and dictatorial manner	−0.347	< 0.001
Expects complete and unquestioning obedience	−0.359	< 0.001
Treats employees harshly and shows little compassion	−0.269	< 0.001
Manipulates others to serve personal interests	−0.250	0.001
Uses charm or charisma primarily for self‐benefit	−0.274	< 0.001
Takes full credit for successes and shifts blame for failures	−0.272	< 0.001

*Note: r* = Pearson correlation coefficient. A negative value means that higher despotic leadership is associated with lower thriving at work.

Table 8 presents the multivariable linear regression analysis examining whether perceived despotic leadership remained associated with thriving at work after adjustment for gender, nursing experience, department/unit, average weekly working hours, and work‐schedule pattern. Perceived despotic leadership remained significantly associated with lower thriving at work (*B* = −0.321, SE = 0.061, *β* = −0.371, 95% CI [−0.441, −0.201], *p* < 0.001). The overall model was statistically significant, *F* (21, 159) = 3.60, *p* < 0.001, and explained 32.2% of the variance in thriving at work (adjusted *R* ^2^ = 0.233).

**TABLE 8:** Multivariable linear regression analysis of thriving at work.

Predictor	*B*	SE	Standardized *β*	95% CI for *B*	*p*‐value
Perceived despotic leadership	**−0.321**	0.061	**−0.371**	−0.441 to −0.201	**< 0.001**

*Note: B* = regression coefficient; *β* = standardized coefficient. The analysis was adjusted for selected demographic and work‐related factors. Bold values indicate statistical significance (*p* < 0.001).

Abbreviations: CI = confidence interval, SE = standard error.

## 4. Discussion

The present study examined the relationship between perceived despotic leadership and thriving at work among nurses at KFMC in Riyadh, Saudi Arabia. The findings revealed that nurses reported a moderate level of perceived despotic leadership and a moderate level of thriving at work. The results also showed a statistically significant negative correlation between despotic leadership and thriving at work, indicating that higher perceptions of despotic leadership were associated with lower levels of thriving among nurses. These findings provide important evidence that leadership style is not only related to negative workplace outcomes but also to nurses’ ability to experience vitality, learning, meaning, and professional growth within the clinical work environment.

The findings indicated that the average score of perceived despotic leadership categorized the level of perceived despotic leadership among the nurses as moderate. The finding shows that the behavior encompassing despotic leadership was not considered highly perceived, thus being present to some extent in the work environment. Among the items rated highest is the item connected with the leader stating his merits and blaming others for the bad results of the work, along with the instance of being charming mainly for the sake of personal interest. This suggests that the nurses consider despotic leadership to be acting on self‐interest principles rather than via a strict despotic approach. This corresponds to the idea of categorization of despotic leadership as being a type of destructive leadership based on self‐interest, power, absence of ethical concerns, and exploitation of the subordinates [8].

Further analysis of individual items adds more detail to the findings. While the item “taking credit for successes and laying blame for failure” was the item with the highest mean of descriptive importance, the item that had the highest association with low thriving was “expecting blind and total compliance,” closely followed by “authoritarian and dictatorial” actions. This fact is significant because it means that the most commonly perceived behavior is not the behavior that is associated with thriving. The results of the study indicate that controlling leadership behavior is particularly valuable for organizations when it comes to setting the focus for leadership development; nonetheless, the relationships seem to be merely associative in nature.

This result is consistent with the findings of Akdoğan et al. [7], which indicated that despotic leadership in nursing circles leads to emotional exhaustion, silence at work, and unfavorable reactions by nurses. The current findings also confirm Mrayyan and Abu‐Hantash [23], who stated that the nurses’ impression of clinical nurse managers’ despotism seriously influences their turnover intentions. Also, the research of Abo Baraka et al. [21] underscores that despotic management by the nursing managers will have a negative impact on group cohesion and emotional experiences of nurses. All these results stress that despotic leadership is an important issue in nursing‐related environments, affecting nurses’ private and occupational life.

The structure of the hospital, along with the work environment, contributes to the limited occurrence of authoritarian leadership in this study. The fact that hospitals are organized in hierarchical structures, sometimes extremely busy, and that decisions have to be made immediately provides opportunities for the autocratic type of leadership. If someone in charge uses blame, too much authority, or behaves in other ways benefiting only them, that person’s leadership will be viewed as autocratic by nurses rather than as directive or supportive. Gruessner is in agreement with the opinion expressed here, pointing out that leadership in the area of healthcare should get away from strict, authoritarian‐type leadership approaches and adopt participative and cooperative approaches, which would create a safe workplace and involve employees in participation.

Moderate levels of thriving were reported among nurses who participated in the study. The most highly rated items included “My work adds meaning to my life” and “At work, I am treated with respect,” indicating that nurses feel their jobs are meaningful even when faced with difficulties in their workplace.

This finding supports Porath et al.’s [9] definition of thriving as a positive psychological state, consisting of both vitality and learning in the workplace. This finding is also supported by Da Silva [1], who stated that thriving nursing environments are supported by emotional intelligence, meaningful work, and positive professional relationships. Similarly, Van Rensburg and Isabirye [5] stated that a thrive‐style workplace culture is dependent upon meaningful work, supportive relationships, and conditions that enable employees to feel valued and engaged in their work. Therefore, while the moderate level score indicating that nurses in this study retain positive professional meaning indicates some level of support, it also indicates that the overall work environment may not foster optimal levels of thriving.

This study supports various hypotheses of the COR theory. Research has shown that nurses working under high job demands, high emotional stress, and little supportive leadership lose both psychological and physical resources over time. Once these resources have been depleted, nurses may not be able to continue to have both energy and function positively. According to Demerouti [12], the balance of job demands and what resources are available to the employee impacts their ability to experience well‐being. In addition, Hollebeek et al. [13] stated that stress is experienced when a person is without access to an important resource or is threatened with losing such a resource. In the present study, the low post‐work energy score suggests that the demands of the clinical environment and feelings of unsupportive leadership contribute to the depletion of resources.

The study found that nurses did not report a significant difference in their perception of despotic leaders based on their demographic and professional backgrounds. The similarities in perceptions among the different groups of nurses suggest that how nurses view and interact with despotic leaders does not vary significantly between different demographic/professional groups.

This result is partly consistent with the idea that destructive leadership is mainly shaped by leader behavior and organizational culture rather than by individual demographic characteristics. Abdelaliem and Zeid [24] found that destructive leadership behaviors were related to organizational silence among nurses, indicating that leadership climate can affect nurses broadly within the organization. Similarly, Hassan and Mohamed [25] reported that despotic leadership was linked to work withdrawal behavior and acquiescent silence among clinical nursing staff. The lack of significant demographic differences in the present study may therefore indicate that leadership behavior is experienced collectively within clinical environments.

However, this finding may differ from some studies that reported variations in nurses’ perceptions according to experience, unit type, or work conditions. Such differences may be due to variations in hospital structure, sample characteristics, measurement tools, or managerial systems. In the present study, the absence of significant differences could be related to the shared institutional policies and common leadership structures across the hospital. It may also be due to the use of convenience sampling, which may limit the ability to detect subgroup differences. Therefore, future studies with larger and more balanced samples across units and positions may provide clearer evidence about whether some groups of nurses are more vulnerable to perceiving despotic leadership than others.

The results showed a statistically significant difference between departments or units in how well they all thrive at work; they did not statistically differ between demographic or professional characteristics. Therefore, it appears that the clinical department has a significant impact on how nurses experience success at work. Individual nurses working in different units face varied amounts of workload, patient acuity, emotional demands, team cohesion, managerial support, and availability of resources; therefore, it seems that success is related not only to the individual but also to the aspects of the clinical unit they work in.

The significant difference in thriving according to department is consistent with the argument that workplace context is central to employee well‐being and positive functioning. Nursing units differ in terms of work intensity, patient needs, staffing patterns, emotional exposure, and learning opportunities. For example, nurses in highly specialized or well‐supported units may experience more professional growth and stronger teamwork, while nurses in units with high workload or limited support may experience lower vitality and lower work satisfaction. Ominyi et al. [3] emphasized that nursing autonomy and evidence‐based practice depend on collaboration and supportive work conditions. Therefore, differences between departments may reflect differences in autonomy, collaboration, and resource availability.

This finding also agrees with the SDT perspective. According to this theory, employees are more likely to function effectively when their needs for autonomy, competence, and relatedness are supported. Gagné et al. [15] explained that SDT is useful for understanding future work because it focuses on how work environments can support or frustrate employees’ basic psychological needs. Ryan and Deci [14] also emphasized that autonomy, competence, and relatedness are essential for quality of life and well‐being. In the present study, some departments may provide better conditions for competence development, professional connection, and decision‐making involvement, which may explain differences in thriving at work.

The principal finding of the study was a statistically significant negative association between perceived despotic leadership and thriving at work. Thus, nurses who reported higher perceived despotic leadership also tended to report lower thriving at work. Importantly, this association remained statistically significant in the multivariable model after adjustment for selected demographic and work‐related characteristics. The overall model explained 32.2% of the variance in thriving at work (adjusted *R* ^2^ = 0.233), suggesting that perceived despotic leadership contributed meaningful explanatory information alongside the included covariates. Nevertheless, because the data were collected at a single time point, the direction of the relationship cannot be established, and the findings should not be interpreted as evidence that despotic leadership causes lower thriving.

Previous studies have shown that despotic leadership is associated with negative consequences for employees and nurses [7]. In his study, Akdoğan and colleagues linked perceived despotic leadership to emotional exhaustion and organizational silence in nurses. Mrayyan and Abu‐Hantash [23] revealed that perceived despotic leadership by clinical nurse managers was associated with nurses having greater turnover intention. Finally, Badar et al. [26] revealed that emotional exhaustion mediates the relationship between despotic leadership and turnover intention.

Taken together, these studies are consistent with the present finding that perceived despotic leadership is associated with less favorable employee outcomes. In the current study, however, resource depletion was not measured as a mediator; COR‐based explanations should therefore be regarded as theoretically plausible interpretations rather than demonstrated mechanisms.

The negative relationship between despotic leadership and thriving can also be explained through the nature of thriving itself. Thriving requires energy, learning, professional confidence, and psychological safety. Despotic leadership, however, may create fear, silence, reduced autonomy, and emotional strain. Alshmemri [27] indicated that despotic leadership blocks open communication and drives acquiescent silence in clinical units. Abdelaliem and Zeid [24] also emphasized the association between destructive leadership behaviors and organizational silence among nurses. When nurses are reluctant to speak, ask questions, or participate in decision‐making, their opportunities for learning and professional development become restricted. As a result, their ability to thrive is weakened.

The current finding is also consistent with Abdou and Albohnayh [28], who discussed emotional exhaustion, job insecurity, and coworker support in relation to toxic nursing leadership. Such findings provide a plausible context for interpreting the present association, but the current study did not measure mediation and therefore cannot establish the mechanism through which despotic leadership relates to thriving. Thakur and Srivastava [29] similarly reported associations between despotic leadership and adverse psychological and engagement‐related outcomes outside the nursing context.

From a theoretical standpoint, the findings support the COR. Despotic leadership may act as a source of resource loss by reducing nurses’ sense of control, security, recognition, and emotional energy. When nurses perceive their leaders as self‐serving, controlling, or unfair, they may protect their remaining resources through silence, withdrawal, or reduced psychological investment in work. This process directly conflicts with thriving, which requires active engagement, vitality, and learning. Demerouti [12] emphasized that job demands and resources are central to explaining employee well‐being and future job design. Accordingly, supportive resources and lower levels of destructive leadership may represent relevant organizational conditions to examine in future studies of nurses’ thriving.

The findings are also compatible with SDT. Despotic leadership may be associated with work conditions that frustrate autonomy, competence, and relatedness; however, these psychological needs were not directly tested as mediators in the present study. The observed negative correlation is therefore interpreted as consistent with, rather than proof of, the SDT‐based proposition that supportive work environments are more conducive to positive motivation, belonging, and professional development [16].

These findings have practical relevance for nursing managers and healthcare organizations. The observed negative associations suggest that hospitals may benefit from leadership development approaches that emphasize ethical, supportive, empathetic, and participatory practices. Relevant development areas include communication, fairness, emotional intelligence, constructive feedback, conflict resolution, accountability, and staff empowerment. Such recommendations are consistent with literature emphasizing empathetic and participatory leadership approaches that support psychological safety and staff engagement [2, 4].

The findings highlight the need for tailored interventions at the unit level that can enhance thriving, as noted that thriving varies widely by department and therefore needs to be addressed individually by each nurse manager through an evaluation of all unit‐based elements prior to implementing an intervention. Deviations in scores related to thriving may indicate that the units require additional support, including assessing the workload of nurses, implementing peer support programs, providing resilience training, and developing a formalized structure for sharing feedback regarding the unit. Velasco & Wald [30] identified the impact of psychological capital and coworker support in reducing the negative impact of work environments on workers, especially those working in extremely intense clinical environments.

### 4.1. Limitation

This study has several limitations. First, the cross‐sectional design precludes determination of temporal order or causality; reverse or bidirectional relationships are possible. Second, the study was conducted at a single tertiary hospital in Riyadh, which limits generalizability to other healthcare settings and regions. Third, self‐reported electronic questionnaires may be affected by social desirability, recall, or common‐method bias, particularly when participants evaluate their immediate managers. Fourth, convenience sampling may have introduced selection bias. Although the multivariable regression adjusted for selected demographic and work‐related characteristics, residual confounding remains possible because other relevant organizational and individual factors were not measured or included in the model. Some demographic and professional groups had very few participants, which may have affected the accuracy of the ANOVA results. Therefore, these findings should be interpreted with caution. Further studies with larger and more balanced groups are recommended to confirm the results.

### 4.2. Implications for Nursing Practice

The observed association suggests that ethical and supportive leadership is an important organizational consideration when seeking to foster nurses’ thriving at work. Healthcare organizations may consider leadership development focused on respectful communication, fairness, constructive feedback, staff participation, psychological safety, and professional development. Because this study did not test an intervention or establish causality, these implications should be viewed as practice‐oriented considerations that warrant prospective evaluation.

### 4.3. Recommendations

Healthcare organizations should consider strengthening ethical, supportive, and respectful leadership practices and providing nurse managers with development opportunities in communication, emotional intelligence, fairness, accountability, constructive feedback, and staff participation. Confidential mechanisms for reporting inappropriate leadership behaviors may also be considered. Unit‐level assessments of workload, staffing, recognition, and opportunities for professional growth may help organizations identify conditions associated with lower thriving. These recommendations are informed by the observed associations and should be evaluated through longitudinal and intervention research.

### 4.4. Recommendations for Future Research

Future studies should examine perceived despotic leadership and thriving at work using larger, multicenter, and more diverse samples. Longitudinal designs are needed to establish temporal ordering and to clarify the direction of the observed association. Future adjusted analyses should include broader theory‐driven organizational and individual factors, such as workload, staffing adequacy, psychological safety, organizational support, and leadership climate. Where psychometrically justified, future studies may also examine theoretically meaningful scale domains or item‐level patterns while controlling for multiple testing. Mediation studies could directly test COR‐ and SDT‐informed mechanisms such as resource depletion, autonomy, competence, relatedness, psychological safety, and organizational support. Intervention studies are also needed to determine whether leadership‐development strategies are followed by improvements in nurses’ thriving.

## 5. Conclusion

Among registered nurses in this Saudi tertiary hospital, perceived despotic leadership and thriving at work were both reported at moderate levels, and higher perceived despotic leadership was significantly associated with lower thriving at work. This association remained significant after adjustment for selected demographic and work‐related characteristics. The cross‐sectional design does not permit conclusions about causality or the direction of the relationship. Nevertheless, the findings underscore the relevance of leadership climate to nurses’ work‐related well‐being and support further evaluation of ethical, supportive, and participatory leadership practices. Longitudinal and multicenter studies are needed to clarify temporal relationships, examine additional explanatory factors, and assess whether leadership‐focused interventions are associated with improvements in nurses’ thriving.

**Nomenclature**
COR: Conservation of Resources
DNP: Doctor of Nursing Practice
HSD: Honestly Significant Difference
ICU: Intensive Care Unit
KFMC: King Fahad Medical City
PsyCap: Psychological Capital
SDT: Self‐Determination Theory

## Author Contributions

Shaimaa Mohamed Elghareeb Allam (principal investigator): conceptualization, methodology, supervision, project administration, formal analysis, software, validation, visualization, writing–review and editing, and final approval of the manuscript. Maryam Murdhi Attalh Alanizi: conceptualization, methodology, investigation, data collection, data curation, formal analysis, software, writing–original draft, visualization, and manuscript preparation. Amany Ahmed Abdrbo: methodology, validation, and writing–review and editing. Rasha Awad Abdelmagied: investigation, validation, and writing–review and editing. Sara El Sayed Abd El Atty: investigation, resources, and writing–review and editing. Farida Said Abed El Maksoud Ahmed: resources, supervision, and writing–review and editing. Somaya Ahmed Bayoumy: validation, supervision, and writing–review and editing. Naiema Mohamed Eleseawy Abdullah: formal analysis, validation, and writing–review and editing. Heba Mohammed Mahmoud Elhapashy: supervision, validation, and writing–review and editing.

## Funding

This research received no external funding and was conducted using the authors’ own resources.

## Disclosure

Patients and members of the public were not involved in the design, conduct, reporting, or dissemination plans of this research. The study focused on registered nurses working at King Fahd Medical City, and only eligible nursing staff participated in the completion of the study questionnaire.

## Ethics Statement

Institutional Review Board (IRB) of the Executive Administration of Nursing Affairs at Riyadh Second Health Cluster granted permission for conducting this study (IRB no. 28–81E) before any data collection occurred. Administrative permission had also been sought from King Fahad Medical City (KFMC) before conducting this study. This study adhered to the Guidelines of the Declaration of Helsinki, and all applicable institutional guidelines were followed during data collection. Participation was voluntary, and electronic informed consent was obtained from all participants. All data collected will remain confidential; thus, no personal identifying information was requested from participants. All data collected will be anonymized and maintained in a protected and secure manner and will only be utilized for the purpose of conducting this research. Study findings will be published as aggregate data so that the privacy and confidentiality of all individuals who participated in this study remain intact [31].

## Consent

No identifying photos or participant personal or clinical information that would jeopardize anonymity are included in this study. As a result, specific consent is not needed for publication.

## Conflicts of Interest

The authors declare no conflicts of interest.

## Supporting Information

Additional supporting information can be found online in the Supporting Information section.

## Supporting information


**Supporting Information** STROBE_checklist.

## Data Availability

The data that support the findings of this study are available in the Supporting Information of this article.
